# Aggregation of SND1 in Stress Granules is Associated with the Microtubule Cytoskeleton During Heat Shock Stimulus

**DOI:** 10.1002/ar.23642

**Published:** 2017-07-31

**Authors:** Jie Shao, Fei Gao, Bingbing Zhang, Meng Zhao, Yunli Zhou, Jinyan He, Li Ren, Zhi Yao, Jie Yang, Chao Su, Xingjie Gao

**Affiliations:** ^1^ Department of Clinical Laboratory Tianjin Medical University Cancer Institute & Hospital Tianjin 300060 People's Republic of China; ^2^ Department of Pediatric Cardiology Tianjin Children's Hospital Tianjin 300070 People's Republic of China; ^3^ Department of Immunology Basic Medical College, Tianjin Medical University Tianjin 300070 People's Republic of China; ^4^ Key Laboratory of Educational Ministry of China Tianjin 300070 People's Republic of China; ^5^ Department of Physiology Basic Medical College, Tianjin Medical University Tianjin 300070 People's Republic of China; ^6^ Laboratory of Molecular Immunology Research Center of Basic Medical Science, Tianjin Medical University Tianjin 300070 People's Republic of China

**Keywords:** SND1, stress granules, microtubule, heat shock

## Abstract

Stress granules (SGs) are dynamic dense structures in the cytoplasm that form in response to a variety of environmental stress stimuli. Staphylococcal nuclease and Tudor domain containing 1 (SND1) is a type of RNA‐binding protein and has been identified as a transcriptional co‐activator. Our previous studies have shown that SND1 is a component of the stress granule, which forms under stress conditions. Here, we observed that SND1 granules were often surrounded by ɑ‐tubulin‐microtubules in 45°C‐treated HeLa cells at 15 min or colocalized with microtubules at 30 or 45 min. Furthermore, Nocodazole‐mediated microtubule depolymerization could significantly affect the efficient recruitment of SND1 proteins to the SGs during heat shock stress. In addition, the 45°C heat shock mediated the enhancement of eIF2α phosphorylation, which was not affected by treatment with Nocodazole, an agent that disrupts the cytoskeleton. The intact microtubule cytoskeletal tracks are important for the efficient assembly of SND1 granules under heat shock stress and may facilitate SND1 shuttling between cytoplasmic RNA foci. Anat Rec, 300:2192–2199, 2017. © 2017 The Authors The Anatomical Record published by Wiley Periodicals, Inc. on behalf of American Association of Anatomists.

Cells either activate defense mechanisms to survive or initiate cell death mechanisms, such as apoptosis, when confronted with unfavorable stress (Fulda et al., 2010). Several stressors (e.g., toxic arsenite or heat shock) can induce the assembly of stress granules (SGs) (Nostramo and Herman, 2016). SGs contain stalled translation preinitiation complexes, which limit the aggregation of misfolded proteins and mRNA damage (Arimoto‐Matsuzaki et al., 2016; Panas et al., 2016). Assembly of SGs requires RNA‐binding proteins, such as TIA‐1/TIAR, or the Ras‐GAP SH3 domain‐binding protein G3BP and can be triggered by stress‐induced phosphorylation of eIF2α (Ser 51), which prevents the formation of the eIF2‐GTP‐Met tRNAi initiation complex (Anderson and Kedersha, 2008). SGs are composed of mRNAs in conjunction with a subset of translation initiation factors, the 40S ribosomal subunit, and RNA binding proteins, and SGs are associated with several clinical diseases (Anderson and Kedersha, 2008; Protter and Parker, 2016; Sfakianos et al., 2016).

The staphylococcal nuclease and Tudor domain containing 1 (SND1) protein was first identified as a co‐activator of Epstein‐Barr virus nuclear protein 2 (EBNA2) (Tong et al., 1995) and was subsequently discovered as a coregulator of both the signal transducer and activator of transcription 6 (STAT6) transcription factor in IL‐4‐mediated gene regulation (Yang et al., 2002) and STAT5 in the prolactin (PRL) signaling pathway (Paukku et al., 2003). SND1 can also be co‐purified with the U5 small nuclear ribonucleoprotein (snRNP) complex and promote spliceosome assembly *in vitro* (Yang et al., 2007). Interestingly, SND1 is an integral part of the RNA‐induced silencing complex (RISC) (Scadden, 2005) and can recognize hyper‐edited double‐stranded RNAs (I‐dsRNAs), which specifically bind to several SG components (Scadden, 2007). Furthermore, SND1 was identified as a component of the SGs of plant and animal cells (Gao et al., 2010; Weissbach and Scadden, 2012; Yan et al., 2014; Gutierrez‐Beltran et al., 2015). However, further data with respect to the molecular mechanism underlying the role of SND1 in SG assembly are needed. In the present study, we focused on the relationship between the microtubule cytoskeleton and SND1‐containing SGs during heat shock stimulation and found that the SND1 protein aggregated into SGs in a microtubule‐dependent manner.

## MATERIALS AND METHODS

### Cells Line, Plasmids, and Transfections

HeLa cells were purchased from the American Type Culture Collection and cultured in Dulbecco's minimal essential medium (Invitrogen Life Technologies, Madrid, Spain) containing 10% fetal bovine serum. A plasmid encoding RFP epitope‐tagged SND1 (RFP‐SND1) was generated as previously described (Gao et al., 2010). Transfections of HeLa cells were performed using L‐polyethyleneimine (Sigma–Aldrich, St Louis, MO), according to the manufacturer's instructions.

### Immunofluorescence

Immunofluorescence assays were conducted as previously described (Gao et al., 2010). Cells were untreated or treated with a 45°C heat shock for different times (0 min, 5 min, 15 min, 30 min, and 45 min). After fixation and permeabilization, cells were incubated with goat anti‐SND1 (Santa Cruz Biotechnology, Santa Cruz, CA) and rabbit anti‐α‐Tubulin (Abcam, Cambridge, UK) antibodies. Then, the Texas Red 596‐coupled anti‐goat polyclonal secondary antibodies to goat IgG H&L (Abcam) and Alexa Fluor 488‐coupled antibodies against rabbit IgG (Molecular Probes) were added for the fluorescence signal. The images and the fluorescence intensity profiles of the targeted regions were obtained with a Zeiss confocal microscope (Germany). At least 50 cells were examined.

In addition, for the Nocodazole treatment experiments, HeLa cells were pretreated with 2 μg mL^−1^ Nocodazole for 2 h and stimulated with or without the 45°C heat shock for 45 min. Immunofluorescence was performed using the rabbit anti‐SND1 (ab65078, Abcam) and mouse anti‐α‐Tubulin (T5168, Sigma) primary antibodies. After the fluorescent staining with Alexa Fluor 488‐coupled antibodies against rabbit IgG (Molecular Probes) and Alexa Fluor 546‐coupled anti‐mouse IgG antibody (Molecular Probes), an inverted Leica DMI6000 B microscope was used to collect the images. At least 50 cells were examined.

### Western Blot Analysis

Western blot analysis was conducted as previously described (Gao et al., 2010; Su et al., 2015). Briefly, HeLa cells were treated or untreated at 45°C for 45 min after the pretreatment with or without 2 μg mL^−1^ Nocodazole for 2 h. The total cell lysates were harvested and subjected to SDS‐PAGE. The following antibodies were used: rabbit anti‐SND1 (ab65078, Abcam), mouse anti‐α‐Tubulin (T5168, Sigma), mouse monoclonal anti‐β‐actin antibody (A1978, Sigma Aldrich), rabbit anti‐eIF2α (#5324; Cell Signaling Technology, Beverly, MA) and rabbit anti‐p‐eIF2α (Ser 51) (#3597; Cell Signaling Technology). The ImageJ 2X software (NIMH, Bethesda, MD) was used to measure the grayscale value of the band. The level of p‐eIF2α (Ser 51) was normalized to the total eIF2α protein.

## RESULTS

### Colocalization of SND1 and ɑ‐Tubulin‐Marked Microtubules During Heat Shock

**Figure 1:**
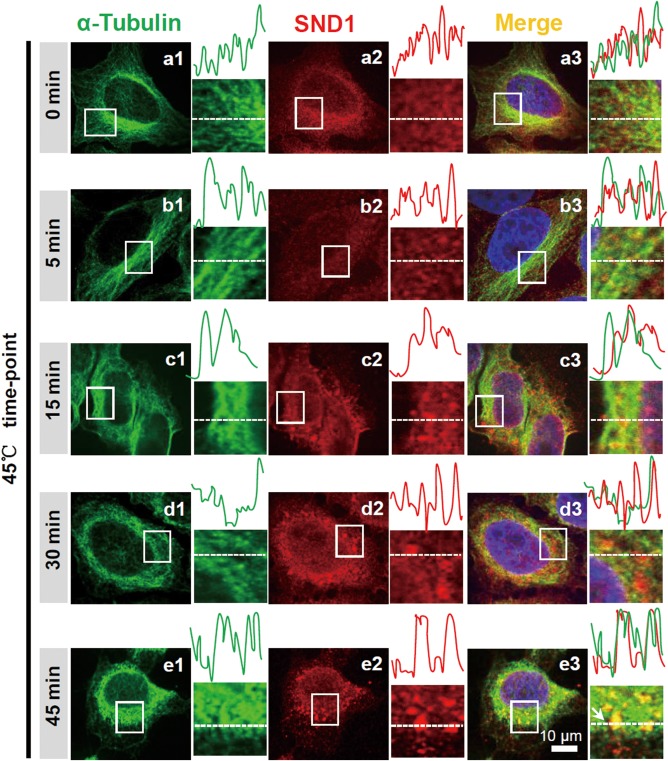
Colocalization of the endogenous SND1 protein and ɑ‐tubulin‐marked microtubules under heat shock stress. HeLa cells were treated at 45°C for 0 min, 5 min, 15 min, 30 min, and 45 min. Then, the endogenous SND1 and ɑ‐tubulin proteins were stained with the anti‐SND1 and anti‐ɑ‐tubulin antibodies, followed by Texas Red 596 or Alexa 488‐conjugated secondary antibodies. Images were obtained by Zeiss confocal microscopy. Fluorescence intensity profiles of regions indicated by short dashed lines are also shown. Scale bar, 10 μm.

To investigate whether the dynamic recruitment of endogenous SND1 into SGs occurred in a microtubule‐dependent manner, we performed an immunofluorescence assay under a heat shock stimulus at different time‐points. HeLa cells were cultured and stimulated with 45°C for 0 min, 5 min, 15 min, 30 min, and 45 min. The fluorescence signals of SND1 and α‐tubulin‐marked microtubules were detected with anti‐SND1 and anti‐ɑ‐tubulin antibodies, followed by Texas Red 596 or Alexa 488‐conjugated secondary antibodies. Fluorescence intensity profiles were used to quantify the degree of co‐localization. As shown in Figure 1, compared with the 0‐min time point (a1‐a3 normal control), there were very few SND1 granules (b2, <10%), and short‐term stress had almost no effect on the microtubules (b1) when cells were stimulated for 5 min. As the stress time increased, the SND1 proteins became aggregated into the granule structure (c2, d2, e2), and the distribution of α‐Tubulin gradually changed from a clear filamentous distribution to an unclear irregular aggregated state (a1‐e1). At 15 min of stimulation, several SND1 granules (10–50%) were surrounded by microtubules (c1‐c3). At 30 min and 45 min, more SND1 granules (>50%) were colocalized with the microtubules (d1‐d3, e1‐e3).

**Figure 2:**
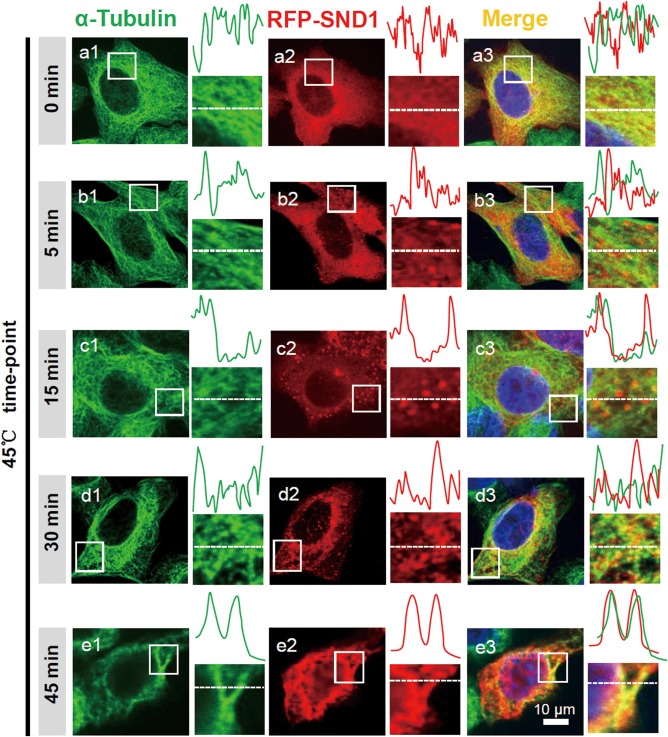
Colocalization of the RFP‐SND1 protein and ɑ‐tubulin‐marked microtubules under heat shock stress. HeLa cells were transfected with the RFP‐SND1 plasmid and treated at 45°C for 0 min, 5 min, 15 min, 30 min, and 45 min. Then, the endogenous ɑ‐tubulin protein was stained with the anti‐ɑ‐tubulin antibody, followed by the Alexa 488‐conjugated secondary antibody. Images were obtained by Zeiss confocal microscopy. Fluorescence intensity profiles of regions indicated by short dashed lines are also shown. Scale bar, 10 μm.

To further analyze the colocalization relationship between exogenous SND1 and the α‐tubulin protein during stress conditions, we transfected the RFP‐SND1 plasmid into HeLa cells and re‐performed the immunofluorescence assay. As shown in Figure 2, similar results were obtained; particularly at 45 min, an obvious co‐localization of SND1 and α‐tubulin was observed (e1‐e3). These observations suggested that the aggregation of SND1 granules may become a part of the scaffold of the microtubule cytoskeleton and slide along the microtubule tracks during heat shock stress.

### The Effect of Nocodazole in the Formation of the SND1 Granule During Heat Shock

**Figure 3:**
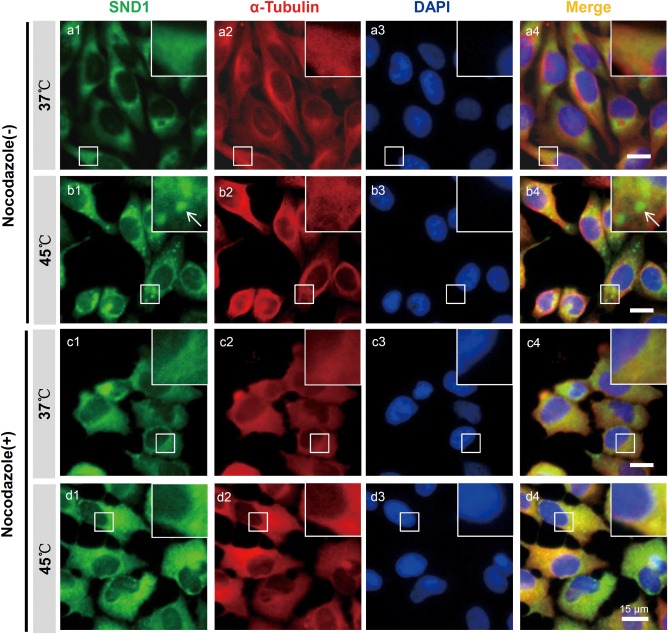
The effect of Nocodazole on SND1 granule formation under heat shock stress. HeLa cells were not pretreated (−) or pretreated with 2 μg mL^−1^ Nocodazole for 2 h (+) and then stimulated with a 45°C heat shock for 45 min or not stimulated (37°C). The Immunofluorescence assay was performed using the rabbit anti‐SND1 and mouse anti‐α‐Tubulin primary antibodies and Alexa Fluor 488‐ or Alexa Fluor 546‐coupled secondary antibodies. An inverted Leica DMI6000 B microscope was used to obtain the images. Arrow: one SND1 granule. Scale bar, 15 μm.

Next, we used 2 μg mL^−1^ Nocodazole to induce depolymerization of the microtubules and detected the aggregation of the SND1 granules in the presence or absence of the 45°C heat shock. As shown in Figure 3, compared with the 37°C group (a1‐a2), heat shock stress induced aggregation of the SND1 granules in the cytoplasm (b1‐b4). However, when the Nocodazole was added, the SND1 granule assembly was impaired (d1‐d4). We failed to observe the efficient formation of SND1 granules when cells were treated with Nocodazole at 37°C (c1‐c4). These results suggested that Nocodazole‐mediated microtubule depolymerization had a strong impact on the recruitment of SND1 proteins to the SGs during the heat shock stress. The cytoskeleton may function as tracks for the accumulation of SND1 proteins into the SGs.

### The Effect of Nocodazole in Heat Shock‐Induced eIF2α Phosphorylation

**Figure 4:**
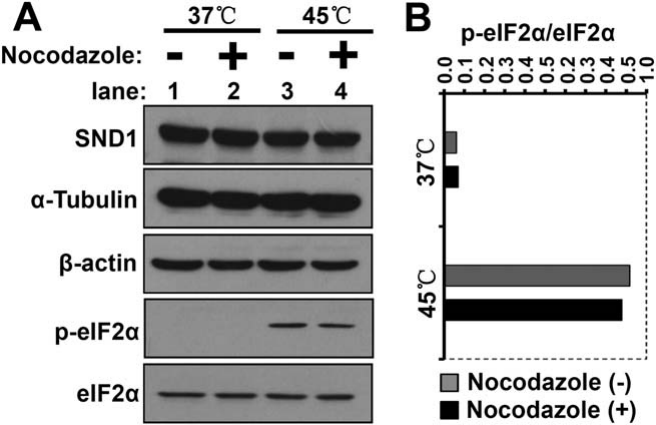
The effect of Nocodazole on heat shock‐induced eIF2α phosphorylation under heat shock stress. (**A**) HeLa cells were untreated (37°C) or treated at 45°C for 45 min after pretreatment with 2 μg mL^−1^ Nocodazole for 2 h (+) or without the pretreatment (−). The total cell lysates were harvested and subjected to SDS‐PAGE. Western blotting was performed using the rabbit anti‐SND1, mouse anti‐α‐Tubulin, mouse monoclonal anti‐β‐actin, rabbit anti‐eIF2α, and rabbit anti‐p‐eIF2α Antibodies. (**B**) The grayscale value of the band was measured by ImageJ 2X. The level of p‐eIF2α (Ser 51) was normalized to the total eIF2α protein.

Considering that phosphorylation of eIF2α at serine 51 (Ser 51) is required for the assembly of SGs (Anderson and Kedersha, 2008), we also performed western blot assays to analyze the effect of Nocodazole in heat shock‐induced eIF2α phosphorylation. As shown in Figure 4A, the Nocodazole treatment at 37°C (lane 2) or 45°C (lane 4) failed to significantly affect the expression of SND1 or the cytoskeletal components, including α‐Tubulin and β‐actin, compared with the control group (lanes 1 and 3). Enhanced phosphorylation of the eIF2α protein was observed when the cells were treated with the 45°C heat shock (Fig. 4A, lanes 3–4), compared with the 37°C control group (Fig. 4A, lanes 1–2). However, there was no difference in the phosphorylation level of the eIF2α protein at 37°C with (Fig. 4A, lane 2) or without (Fig. 4A, lane 1) the Nocodazole pretreatment. We also found that the addition of Nocodazole did not significantly affect the 45°C heat shock‐induced eIF2α phosphorylation level (Fig. 4A, lanes 3–4). The grayscale value of the band was measured, and Figure 4B shows the relative phosphorylation level of eIF2α. These results indicated that eIF2α phosphorylation was not involved in the inhibition of Nocodazole‐mediated microtubule depolymerization in SND1 granule assembly under heat shock stress.

## DISCUSSION

An increasing number of proteins have been reported to be recruited into stress granule structures (Anderson and Kedersha, 2008; Protter and Parker, 2016; Sfakianos et al., 2016). Apart from the potential role of the targeting protein component in SG assembly during stress conditions, the investigators focused their attention on the surroundings of the SGs, such as the liquid droplet or cytoskeletal network (Chernov et al., 2009; Sfakianos et al., 2016). For instance, SGs can be considered as a solid protein dense core, and the aqueous outer shell may take part in the dynamic assembly of SGs during stress conditions (Sfakianos et al., 2016). The SND1 protein in rice and Arabidopsis is associated with the cytoskeleton (Sami‐Subbu et al., 2001; Chuong et al., 2004; Wang et al., 2008). Here, the relationship between the α‐tubulin‐labeled cytoskeleton and SND1‐containing SGs in mammalian cells was examined in response to heat shock stress.

It has been reported that the cytosolic movement activity of poly(A)^+^ binding protein (PABP)‐containing SGs during assembly and disassembly is associated with the disruption of microtubules (Nadezhdina et al., 2010). However, the dynamics of GFP‐PABP influx into SGs might not be associated with microtubule structure, as evidenced by a fluorescence recovery after photobleaching (FRAP) analysis (Nadezhdina et al., 2010). Previously, we found that the SND1 protein can interact and colocalize with PABP1 and regulate the FRAP dynamics of G3BP‐tracing SGs in response to arsenite sodium‐mediated oxidative stress conditions (Gao et al., 2015). Very recently, we found that c‐Jun N‐terminal kinase (JNK) can increase the phosphorylation of SND1 at threonine 103 (T103), which is involved in the efficient aggregation of the SND1 protein into SGs (Su et al., 2017). Here, we found that the assembly of SND1 granules upon heat shock stimulus in HeLa cells occurs in a microtubule transport‐dependent manner. Similar results were also observed in response to another traditional oxidative stress that was induced by arsenite sodium (data not shown). These results were consistent with reports of other SG components (Ivanov et al., 2003; Kolobova et al., 2009; Bartoli et al., 2011). Unlike for α‐tubulin, SG motility was observed to not be linked to actin (Nadezhdina et al., 2010). Several other cytoskeletal proteins, such as dynein heavy chain 1 (DHC1) and Kinesin Family Member 20A (KIF20A), were reported to affect the assembly dynamics of the SG (Loschi et al., 2009; Taniuchi et al., 2014). Understanding the molecular mechanism that underlies the functional links between cytoskeleton‐associated proteins and SND1 granules merits further experiments.

Different localizations of the SND1 protein were observed between animal and plant cells. The SND1 protein in plants was found to be colocalized with SGs and other important functional cytoplasmic foci known as P‐bodies (PBs) (Gutierrez‐Beltran et al., 2015). PB structure has also been reported to be linked to the microtubule (Sweet et al., 2007; Aizer et al., 2008). For example, chemical inhibitor‐mediated microtubule destabilization can induce the aggregation of PBs, independent of the change in mRNA metabolism in budding yeast or *Saccharomyces cerevisiae* (Sweet et al., 2007). The microtubule has been reported to take part in the connection between SGs and PBs (Rajgor and Shanahan, 2014; Rajgor et al., 2014). For instance, Gutierrez‐Beltran et al. reported that microtubule destabilization can influence the formation of SND1‐containing SGs/PBs in Arabidopsis, and the SND1 protein within the PB may function as a key enzymatic component toward regulating the processing of mRNA decapping in response to stress (Gutierrez‐Beltran et al., 2015). Previously, we found that the SND1 protein in HeLa cells can regulate the dynamic aggregation of the *AGTR1 3′UTR* (3′‐untranslated region of angiotensin II receptor, type 1 mRNA) into SGs (Gao et al., 2014) and selectively stabilize SG‐associated mRNA during oxidative stress (Gao et al., 2015). Further evidence is needed to study the relationship between the cytoskeletal network and the role of SND1 granules in mRNA metabolism in different species.

In summary, we have reported the microtubule‐dependent association of SND1 with SGs in animal cells. Emerging evidence with respect to the relationship between clinical diseases and SG assembly has been obtained (Anderson and Kedersha, 2008; Protter and Parker, 2016; Sfakianos et al., 2016). In addition, several chemotherapeutic agents for cancer, such as Darinaparsin (ZIO‐101) and Bortezomib, were reported to influence SG formation or the disruption of microtubules (Fournier et al., 2010; Mason et al., 2011). Considering the association between SND1 and tumorigenesis (Jariwala et al., 2015; Gutierrez‐Beltran et al., 2016), it will be meaningful to further investigate the link between SND1 granules and the cytoskeletal structure, based on several clinical pharmaceuticals or chemotherapeutics.
